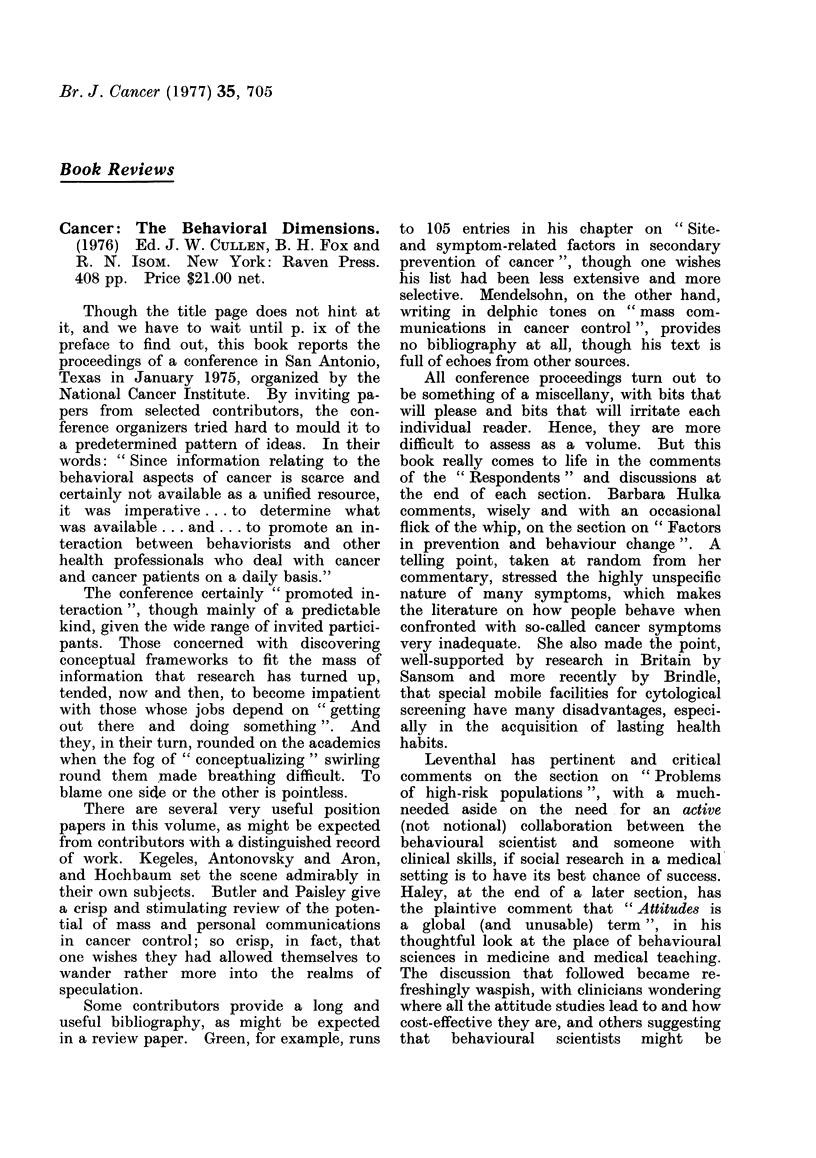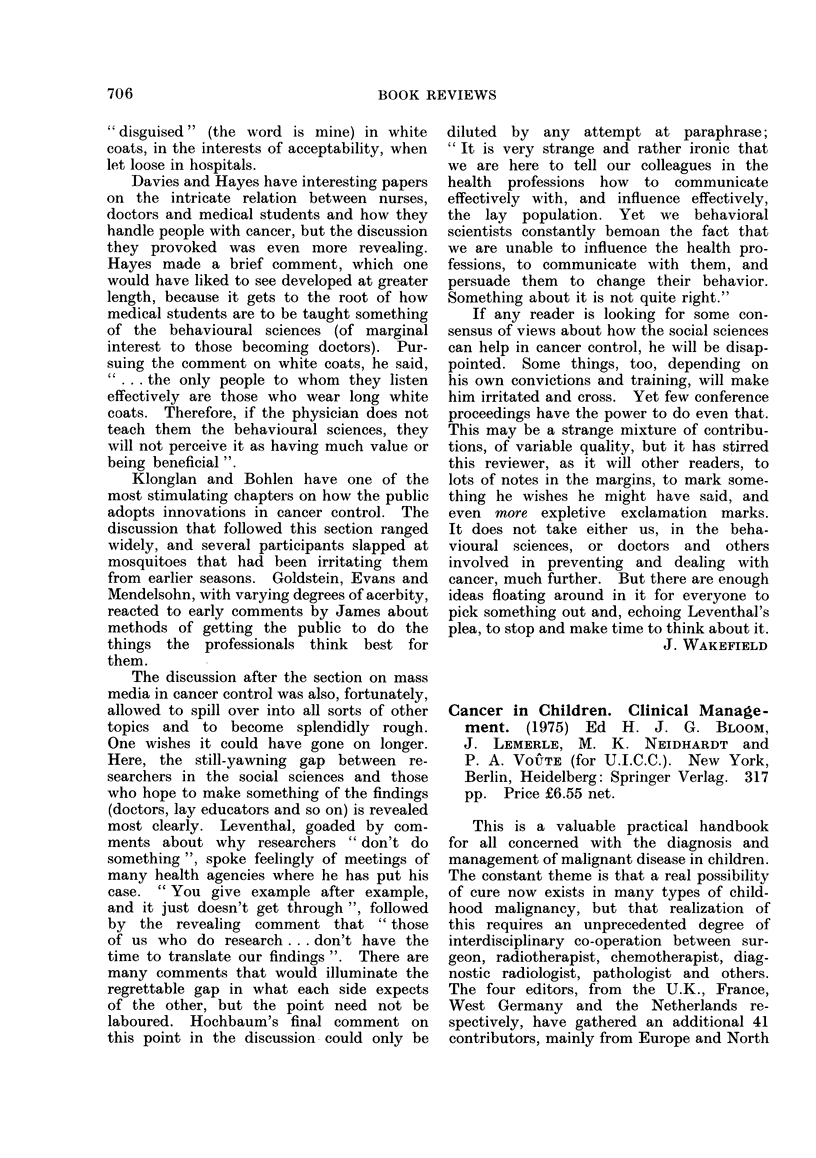# Cancer: The Behavioral Dimensions

**Published:** 1977-05

**Authors:** J. Wakefield


*Br. J. Cancer* (1977) **35**, 705

### *Book Reviews*

**Cancer: The Behavioral Dimensions.** (1976) Ed. J. W. Cullen, B. H. Fox and R. N. Isom. New York: Raven Press. 408 pp. Price $21.00 net.

Though the title page does not hint at it, and we have to wait until p. ix of the preface to find out, this book reports the proceedings of a conference in San Antonio, Texas in January 1975, organized by the National Cancer Institute. By inviting papers from selected contributors, the conference organizers tried hard to mould it to a predetermined pattern of ideas. In their words: " Since information relating to the behavioral aspects of cancer is scarce and certainly not available as a unified resource, it was imperative . . . to determine what was available . . . and . . . to promote an interaction between behaviorists and other health professionals who deal with cancer and cancer patients on a daily basis."

The conference certainly " promoted interaction ", though mainly of a predictable kind, given the wide range of invited participants. Those concerned with discovering conceptual frameworks to fit the mass of information that research has turned up, tended, now and then, to become impatient with those whose jobs depend on " getting out there and doing something ". And they, in their turn, rounded on the academics when the fog of " conceptualizing " swirling round them made breathing difficult. To blame one side or the other is pointless.

There are several very useful position papers in this volume, as might be expected from contributors with a distinguished record of work. Kegeles, Antonovsky and Aron, and Hochbaum set the scene admirably in their own subjects. Butler and Paisley give a crisp and stimulating review of the potential of mass and personal communications in cancer control; so crisp, in fact, that one wishes they had allowed themselves to wander rather more into the realms of speculation.

Some contributors provide a long and useful bibliography, as might be expected in a review paper. Green, for example, runs to 105 entries in his chapter on " Site- and symptom-related factors in secondary prevention of cancer ", though one wishes his list had been less extensive and more selective. Mendelsohn, on the other hand, writing in delphic tones on " mass communications in cancer control ", provides no bibliography at all, though his text is full of echoes from other sources.

All conference proceedings turn out to be something of a miscellany, with bits that will please and bits that will irritate each individual reader. Hence, they are more difficult to assess as a volume. But this book really comes to life in the comments of the " Respondents " and discussions at the end of each section. Barbara Hulka comments, wisely and with an occasional flick of the whip, on the section on " Factors in prevention and behaviour change ". A telling point, taken at random from her commentary, stressed the highly unspecific nature of many symptoms, which makes the literature on how people behave when confronted with so-called cancer symptoms very inadequate. She also made the point, well-supported by research in Britain by Sansom and more recently by Brindle, that special mobile facilities for cytological screening have many disadvantages, especially in the acquisition of lasting health habits.

Leventhal has pertinent and critical comments on the section on " Problems of high-risk populations ", with a much-needed aside on the need for an *active* (not notional) collaboration between the behavioural scientist and someone with clinical skills, if social research in a medical setting is to have its best chance of success. Haley, at the end of a later section, has the plaintive comment that " *Attitudes* is a global (and unusable) term ", in his thoughtful look at the place of behavioural sciences in medicine and medical teaching. The discussion that followed became refreshingly waspish, with clinicians wondering where all the attitude studies lead to and how cost-effective they are, and others suggesting that behavioural scientists might be

"disguised" (the word is mine) in white coats, in the interests of acceptability, when let loose in hospitals.

Davies and Hayes have interesting papers on the intricate relation between nurses, doctors and medical students and how they handle people with cancer, but the discussion they provoked was even more revealing. Hayes made a brief comment, which one would have liked to see developed at greater length, because it gets to the root of how medical students are to be taught something of the behavioural sciences (of marginal interest to those becoming doctors). Pursuing the comment on white coats, he said, " . . . the only people to whom they listen effectively are those who wear long white coats. Therefore, if the physician does not teach them the behavioural sciences, they will not perceive it as having much value or being beneficial ".

Klonglan and Bohlen have one of the most stimulating chapters on how the public adopts innovations in cancer control. The discussion that followed this section ranged widely, and several participants slapped at mosquitoes that had been irritating them from earlier seasons. Goldstein, Evans and Mendelsohn, with varying degrees of acerbity, reacted to early comments by James about methods of getting the public to do the things the professionals think best for them.

The discussion after the section on mass media in cancer control was also, fortunately, allowed to spill over into all sorts of other topics and to become splendidly rough. One wishes it could have gone on longer. Here, the still-yawning gap between researchers in the social sciences and those who hope to make something of the findings (doctors, lay educators and so on) is revealed most clearly. Leventhal, goaded by comments about why researchers " don't do something ", spoke feelingly of meetings of many health agencies where he has put his case. " You give example after example, and it just doesn't get through ", followed by the revealing comment that " those of us who do research . . . don't have the time to translate our findings ". There are many comments that would illuminate the regrettable gap in what each side expects of the other, but the point need not be laboured. Hochbaum's final comment on this point in the discussion could only be diluted by any attempt at paraphrase; " It is very strange and rather ironic that we are here to tell our colleagues in the health professions how to communicate effectively with, and influence effectively, the lay population. Yet we behavioral scientists constantly bemoan the fact that we are unable to influence the health professions, to communicate with them, and persuade them to change their behavior. Something about it is not quite right."

If any reader is looking for some consensus of views about how the social sciences can help in cancer control, he will be disappointed. Some things, too, depending on his own convictions and training, will make him irritated and cross. Yet few conference proceedings have the power to do even that. This may be a strange mixture of contributions, of variable quality, but it has stirred this reviewer, as it will other readers, to lots of notes in the margins, to mark something he wishes he might have said, and even *more* expletive exclamation marks. It does not take either us, in the behavioural sciences, or doctors and others involved in preventing and dealing with cancer, much further. But there are enough ideas floating around in it for everyone to pick something out and, echoing Leventhal's plea, to stop and make time to think about it.

J. WAKEFIELD